# Mitral valve repair and replacement in infectious endocarditis: a systematic review and meta-analysis of clinical outcome

**DOI:** 10.1186/s43044-024-00564-5

**Published:** 2024-10-04

**Authors:** Umesh Nuthalapati, Manoj Reddy Bathinapattla, Rayner Peyser Cardoso, Nusrat Jahan Jesi, Kanwarmandeep Singh, Iman Moradi, Karol Gostomczyk, Maham Afzal, Moosa Bin Omer, Zorez Rashid Mian, Soham Patel, Pratyush Sachdeva, Muhammad Nauman Malik, Mohammad Abbas, Jugraj Singh, Muhammad Ashir Shafique

**Affiliations:** 1 Ivano Frankivsk National Medical University, Ivano-Frankivsk, Ukraine; 2 AIIMS Jodhpur, Jodhpur, India; 3 Shaheed Syed Nazrul Islam Medical College and Hospital, Kishoregonj, Bangladesh; 4 School of Medicine, St. Georges’ University, St. George’s, Grenada; 5 Collegium Medicum Nicolaus Copernicus University, Bydgoszcz, Poland; 6 Government Medical College Amritsar, Amritsar, Punjab India; 7 Fatima Jinnah Medical University, Lahore, Pakistan; 8 CMH Lahore Medical College & IOD, Lahore, Pakistan; 9 Teaching University Geomedi, Tbilisi, Georgia; 10 Punjab Institute of Medical Sciences, Jalandhar, India; 11 Bahawal Victoria Hospital, Bahawalpur, Pakistan; 12 CMH Lahore Medical College, Lahore, Pakistan; 13 Verde Valley Medical Center, Cottonwood, AZ 86326 USA; 14 Department of Medicine, Jinnah Sindh Medical University, Rafiqi H J Shaheed Road, Karachi, 75510 Pakistan

**Keywords:** Infective endocarditis, Mitral valve repair, Mitral valve replacement, Meta-analysis, Outcomes

## Abstract

**Background:**

Infective endocarditis (IE) poses significant clinical challenges, often necessitating surgical intervention for improved patient outcomes. The choice between mitral valve repair (MVP) and mitral valve replacement (MVR) is crucial in managing IE. This systematic review and meta-analysis aims to compare the effectiveness of MVP and MVR in treating IE, focusing on outcomes such as postoperative bleeding, mortality, recurrent endocarditis, and stroke.

**Main text:**

A comprehensive literature search was conducted following PRISMA guidelines. Studies directly comparing MVP and MVR in IE patients were included. Data extraction and quality assessment were performed, and meta-analysis was conducted using RevMan software. Thirty-two studies involving 82,123 patients were included. MVP was associated with significantly lower rates of postoperative bleeding (OR: 0.58, 95% CI: 0.40–0.84) and reduced long-term mortality (OR: 0.40, 95% CI: 0.32–0.51) compared to MVR. However, MVR showed lower rates of recurrent endocarditis. MVP was also associated with a decreased likelihood of postoperative stroke (OR: 0.52, 95% CI: 0.40–0.68).2, 4

**Conclusions:**

MVP demonstrates advantages over MVR in reducing postoperative bleeding, long-term mortality, and stroke risk in IE patients. However, individual patient factors and surgical expertise must be considered in treatment decisions. Further research, including randomized controlled trials, is needed to validate these findings and refine treatment algorithms for IE management.

**Supplementary Information:**

The online version contains supplementary material available at 10.1186/s43044-024-00564-5.

## Background

Endocarditis, an inflammation of the heart's inner lining and valves, is primarily caused by infectious organisms, with Staphylococcus aureus, streptococci, and enterococci collectively responsible for 80–90% of cases in developed nations [1, 2]. The mitral valve is especially susceptible, leading to complications such as stroke, heart failure, and intracardiac abscess. Oropharyngeal bacteria and fungi also contribute to infectious endocarditis [2, 3]. While rare in developed countries, it affects 3–8 per 100,000 people in developing regions, with higher prevalence in males and those over 65 [4]. Risk factors include pre-existing valvular disease, prosthetic valves, diabetes mellitus, hemodialysis, and intravenous drug use [5–7]. Diagnosing IE necessitates a comprehensive approach, encompassing physical examination, history taking, echocardiography, and microbiological tests8, as addressed by the Modified Duke's criteria [8, 9]. Treatment involves empirical and resistance-guided antibiotics, with surgical intervention when necessary [10–12], particularly for complicated cases with unmanageable mitral valve issues [13, 14] or life-threatening complications.

Surgery is imperative in native valve endocarditis with recurrent or persistent emboli or vegetations, particularly when they exceed 10 mm in size [15, 16]. The standard approach to mitral valve repair involves full cardiopulmonary bypass and ischemic arrest with various surgical techniques, such as median sternotomy, right thoracotomy, and robotic assistance [16, 17]. Embracing minimally invasive mitral valve surgery, including partial sternotomy and right thoracotomy, offers notable advantages, such as reduced blood loss, diminished need for blood transfusion, and shorter stays in the intensive care unit (ICU) [18–20].

Partial sternotomy and right thoracotomy, known for lessening blood loss, transfusion requirements, and ICU durations in mitral valve repairs, demand cautious evaluation for IE due to severe valve damage, abscesses, and complex pathologies that may call for more extensive surgery [21–23]. The constrained access of these minimally invasive methods might not suffice for thorough debridement and reconstruction. Furthermore, the presence of infected tissue can hinder the repair process and affect outcomes. Limited and inconclusive research exists on the efficacy of these techniques in IE, suggesting that while they can be effective with careful patient selection and skilled surgery, they might not be appropriate for patients with more severe conditions [24, 25]. Although mitral valve repair is generally favored over replacement for degenerative conditions, its advantage in treating IE is uncertain [26, 27]. In IE cases, the primary goal is to remove infected tissue, which can limit repair options and raise concerns about the long-term durability of complex repairs, particularly in severely infected areas like the annulus or multiple leaflet segments. Some studies report better survival outcomes with mitral valve repair compared to replacement in IE surgery patients, but these studies often involve small cohorts and are usually conducted at a single center [28–30]. In the context of IE, MVP may be constrained by the difficulty of completely removing infected tissue, potentially increasing the likelihood of recurrence. Conversely, MVR, while ensuring the elimination of all infected regions, could result in higher long-term mortality rates and complications associated with the prosthetic valve [29, 31].

Our systematic review and meta-analysis sought to offer a thorough evaluation of MVP and MVR outcomes in managing IE. Despite numerous studies exploring these surgical approaches, a detailed, current analysis incorporating the latest evidence was lacking. The publication of several new studies since the most recent meta-analysis on this subject necessitated an updated review to reflect the most current information [30, 32, 33]. Our research addresses this knowledge gap by delivering a comprehensive analysis that presents the highest level of evidence, thus enhancing and modernizing the existing body of literature on this crucial clinical decision-making process.

## Methods

This meta-analysis was conducted according to the Preferred Reporting Items for Systematic Review and Meta-Analysis (PRISMA) guidelines.

### Literature search and search strategy

We thoroughly searched databases including PubMed, Medline, Google scholar, Scopus, and Embase and identified all studies, and we utilized the terms “Infective endocarditis” AND [MVP OR Mitral Valve Plasty OR mitral valve repair OR mitral valve annuloplasty OR mitral reconstruction] AND [MVR OR mitral valve replacement] as key words for MeSH terms. No language restrictions were applied. The reference lists of relevant articles and reviews were assessed systematically, and inclusion and exclusion criteria were applied.

### Eligibility criteria

The criteria for inclusion in the analysis were as follows: a direct comparison between MVP and MVR in patients with infectious endocarditis, and an evaluation of clinical outcomes such as early and long-term survival, event-free survival, and reinfarction events. Studies were excluded if they did not provide outcome data, had insufficient information, were non-comparative studies of mitral valve repair versus replacement, or involved patients with prosthetic valve endocarditis.

### Data extraction and outcome measures

Two researchers, M. R. and U. N., individually gathered crucial information from the selected studies by examining their titles and abstracts. A third party, M. A. S., was consulted to resolve any disagreements or controversies. They focused on studies that directly contrasted these interventions and provided comprehensive clinical outcome measures, such as mortality rate, postoperative bleeding, and IE recurrence of IE. The collected data encompassed various aspects, including the study title, publication year, first author's name, year of study, country of origin, number of participants, and outcome measures assessed. After the extraction, the data were screened for duplicate studies. The quality of the papers was thoroughly assessed using I.M. to guarantee the validity of our conclusions.

### Data analysis

The Cochrane Collaboration Review Manager (RevMan) software was used for data analysis. For dichotomous data, the estimated effect measures were reported as odds ratio (OR) with 95% confidence intervals (CIs). Statistical significance was determined by a point estimate of the odds ratio at *P* < 0.05, with a 95% CI that did not include the value of one. For continuous data, the weighted mean difference (WMD) was used with the same criteria for statistical significance. The random-effects model was applied in this meta-analysis, considering the point estimates, variance, and weights for each study. The I^2^ statistic was used to assess heterogeneity, with significance considered if the I^2^ exceeded 50%. Heterogeneity was then interpreted based on the characteristics of the study.

### Risk of bias and quality assessment

M.A. and Z.R.M. evaluated the quality of observational studies using the Newcastle–Ottawa quality assessment scale, considering biases in selection, comparability, and outcomes. A score of 7 out of 9 for each study was deemed indicative of low bias. Any challenges encountered were resolved with the assistance of a third researcher, M.A.S. Additionally, publication bias was assessed using Funnel plots provided in Supplementary File. Supplementary Table 1 displays the quality assessment results for the observational studies.

## Results

### Literature Search and screening

**Fig. 1:**
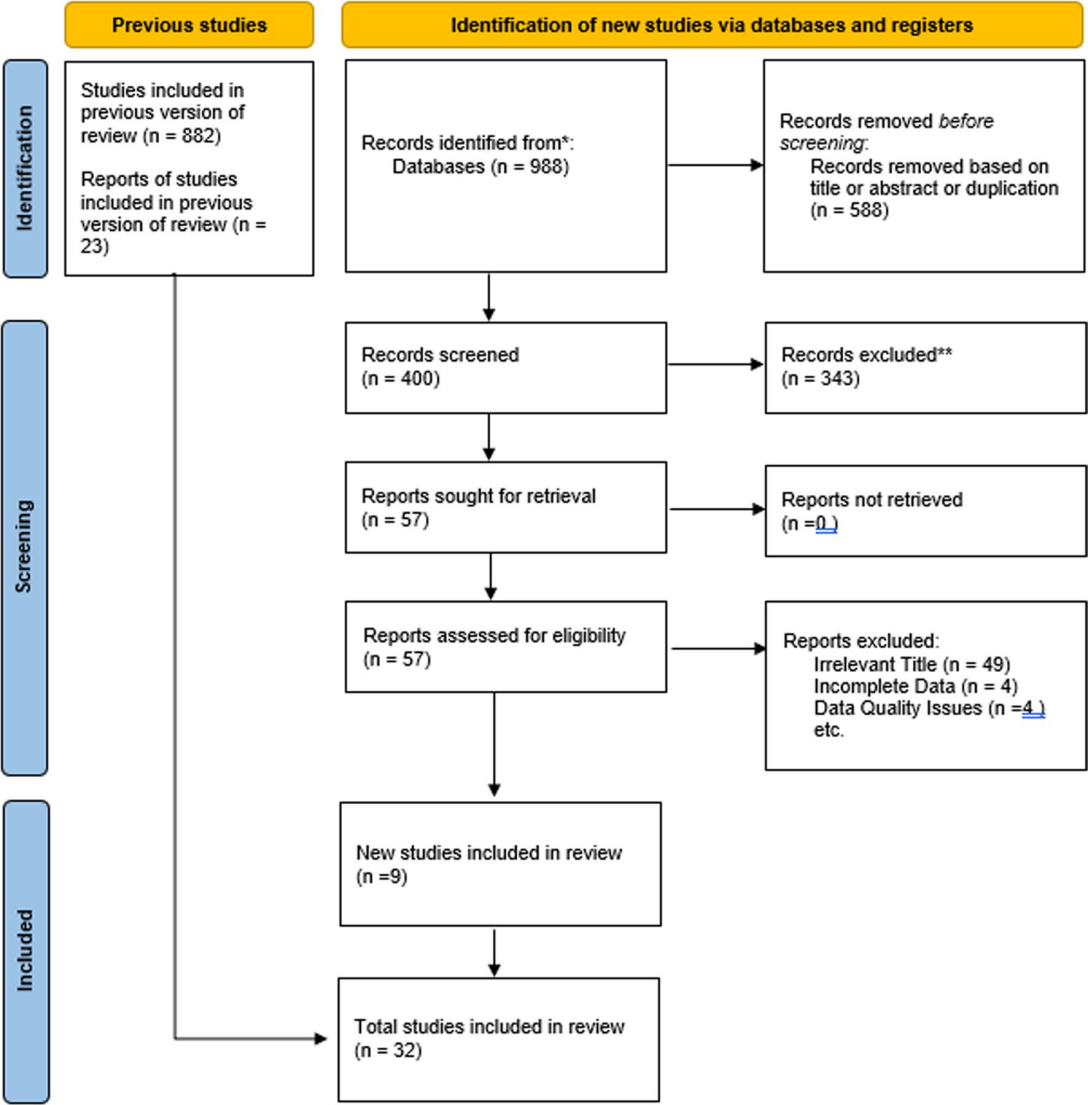
PRISMA flow chart

The selection protocol is illustrated in Fig. 1. Thirty-two studies met the inclusion criteria yielding 82,123 patients in the final analysis [24, 25, 30, 32, 34–60]. The number of studies included ranged from 1997 to 2021. Table 1 further characterizes the individual studies. Postoperative bleeding, mortality, recurrent endocarditis, and stroke were analyzed. The study provides a detailed comparison of MVP versus MVR in patients with IE. The included studies vary in size, with some involving fewer than 1000 patients and others exceeding 1000. The research also differentiates between outcomes for biological versus mechanical valves, particularly addressing how mechanical valves influence bleeding rates. The distribution of patients across these studies and the types of valves used are crucial for understanding the overall efficacy and safety of each surgical approach.

**Table 1:** Baseline characteristics of included studies

Author	Country	Study type	Study period	Surgery		Active/healed	Age in years(mean)		Male population%	
				MVP	MVR		MVP	MVR	MVP	MVR
Bacco et al. [39]	Italy	Observational	2004–2019	53	56	Active	57.7 ± 15.7	60 ± 15.2	75.50%	60.70%
Alkhouli et al. [ 35]	USA	Observational	2003–2016	7451	27,204	Both	53	55	67.10%	60.70%
Feringa et al. [ 40]	Netherlands	Observational	1980–2005	470	724	Active	50.7 ± 8.4	49.5 ± 9.1	74.30%	74.20%
Li et al. [44]	Taiwan	Observational	2000–2013	424	1575	Active	49.3 ± 16.1	52.4 ± 15.8	69.10%	66.30%
Tepsuwan et al. [55]	Thailand	Observational	2006–2017	38	76	Active	44.1 ± 15.8	47.5 ± 15.0	55.30%	75%
Malvindi et al. [25]	Italy	Observational	2001–2021	96	186	Active	60 ± 15		75%	
Ling et al. [50]	UK	Observational	2005–2021	85	98	Both	56	60	67.10%	65.30%
Zubaidi et al. [34]	UK	Observational	2000–2019	1371	3231	Both	66.85	65.31	66.40%	45.80%
Chang et al. [36]	South Korea	Observational	2004–2011	11	15	Both	38.2	48.3	9/2	11/4
Musci et al. [48]	Germany	Observational	1996–2007	61	166	Active	47.7	56.2	40/21	134/85
Wilhelm et al. [59]	Switzerland	Observational	1980–1996	57	97	Both				
Mihaljevic et al. [45]	USA	Observational	1992–2002	21	32	Active	66	50	16	24
Sternik et al. [54]	USA	Observational	1986–1999	16	28	Active				
Tepsuwan et al. [55]	Thailand	Observational	2006–2017	38	76	Active	44.1	47.5	21	57
Shang et al. [53]	USA	Observational	2002–2007	56	33	Both	48		59	
Muehrcke et al. [47]	USA	Observational	1985–1995	102	44	Both	51.9		100	
Miura et al. [46]	Japan	Observational	1985–2012	36	21	Active	57	55	35	27
Lee et al. [43]	Taiwan	Observational	2005–2015	38	33	Active	42.3	53.7	24	20
Tomsic et al. [56]	Netherlands	Observational	2000–2016	51	32	Active	53	60	38	17
Gammie et al. [41]	USA	Observational	1994–2003	1882	4565	Both	56		3956	
Solari et al. [24]	Belgium	Observational	1991–2015	155	37	Active	60.1	64.6	109	18
Perrota et al. [51]	Sweden	Observational	2000–2015	76	64	Both	60	62	55	45
Defauw et al. [38]	Netherlands	Observational	2000–2017	97	53	Active	57	61	20	22
Cuerpo et al. [37]	Spain	Observational	2008–2016	68	301	Active				
Navia et al. [49]	USA	Observational	1988–2017	52	86	Both	55	58	44	52
Wang et al. [58]	New Zealand	Observational	2005–2011	25	35	Active	43.1	52.1	14	21
Yamaguchi et al. [60]	Japan	Observational	1999 -2005	14	7	Both	58		53	
Jung et al. [42]	South Korea	Observational	1994–2009	41	61	Active	34.4	43.1	19	33
Toyoda et al. [57]	USA	Observational	1998–2014	367	1603	Active	56.9	54.9	243	900
Ruttmann et al. [52]	Austria	Observational	1992–2004	34	34	Active	51.5	53.2	22	17

MVP: mitral valve repair; MVR: mitral valve replacement

### Postoperative bleeding

**Fig. 2:**
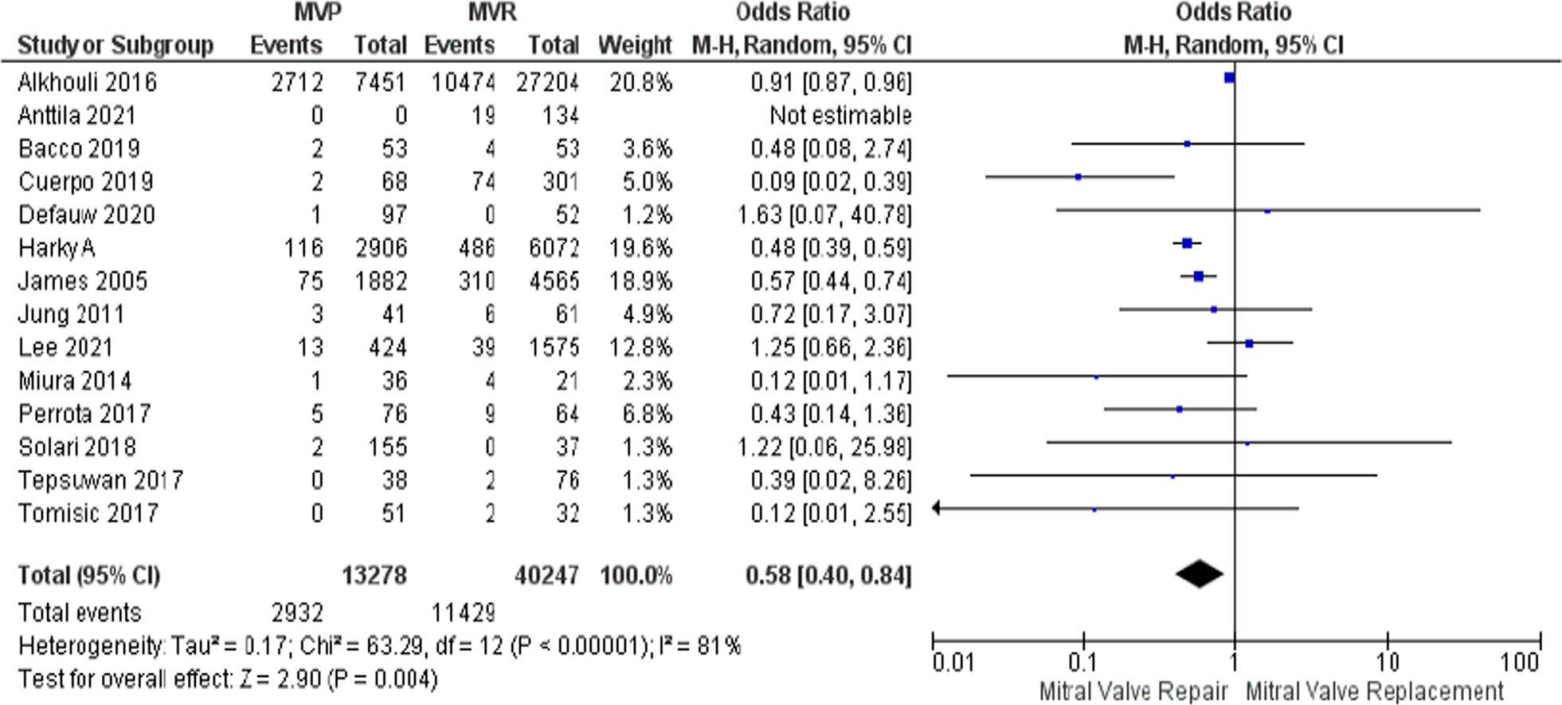
Forest plot for outcome of post-op bleeding rate

The occurrence of postoperative bleeding, which refers to significant bleeding occurring within 30 days after the surgery, was assessed across 32 studies. However, one study by Antilla et al. was excluded due to an inestimable odds ratio (OR). After excluding this study, 31 studies were included in the analysis. These studies provided relevant data on postoperative bleeding for 12 out of the 31 studies, encompassing a total of 16,871 patients. The random effects model used in the analysis revealed that MVP was associated with significantly lower rates of postoperative bleeding compared to MVR. Specifically, the odds ratio for MVP vs MVR was 0.58, with a 95% confidence interval (CI) of 0.40–0.84 and a *p*-value of 0.004, and an *I*^2^ of 81% (Fig. 2). A sensitivity analysis was performed to address heterogeneity, and the results were still significant even when the leave-one-out method was used to exclude influential studies, yielding an odds ratio of 0.50, with a 95% confidence interval of 0.42–0.59 and a *p*-value of 0.00001 (Supplementary Figure S5). Notably, two studies conducted by Harky et al. and James et al. accounted for 93% of the patients included in the analysis. Even when either or both of these studies were excluded, the lower rates of postoperative bleeding observed in patients who underwent MVP remained consistent.

### Mortality

**Fig. 3:**
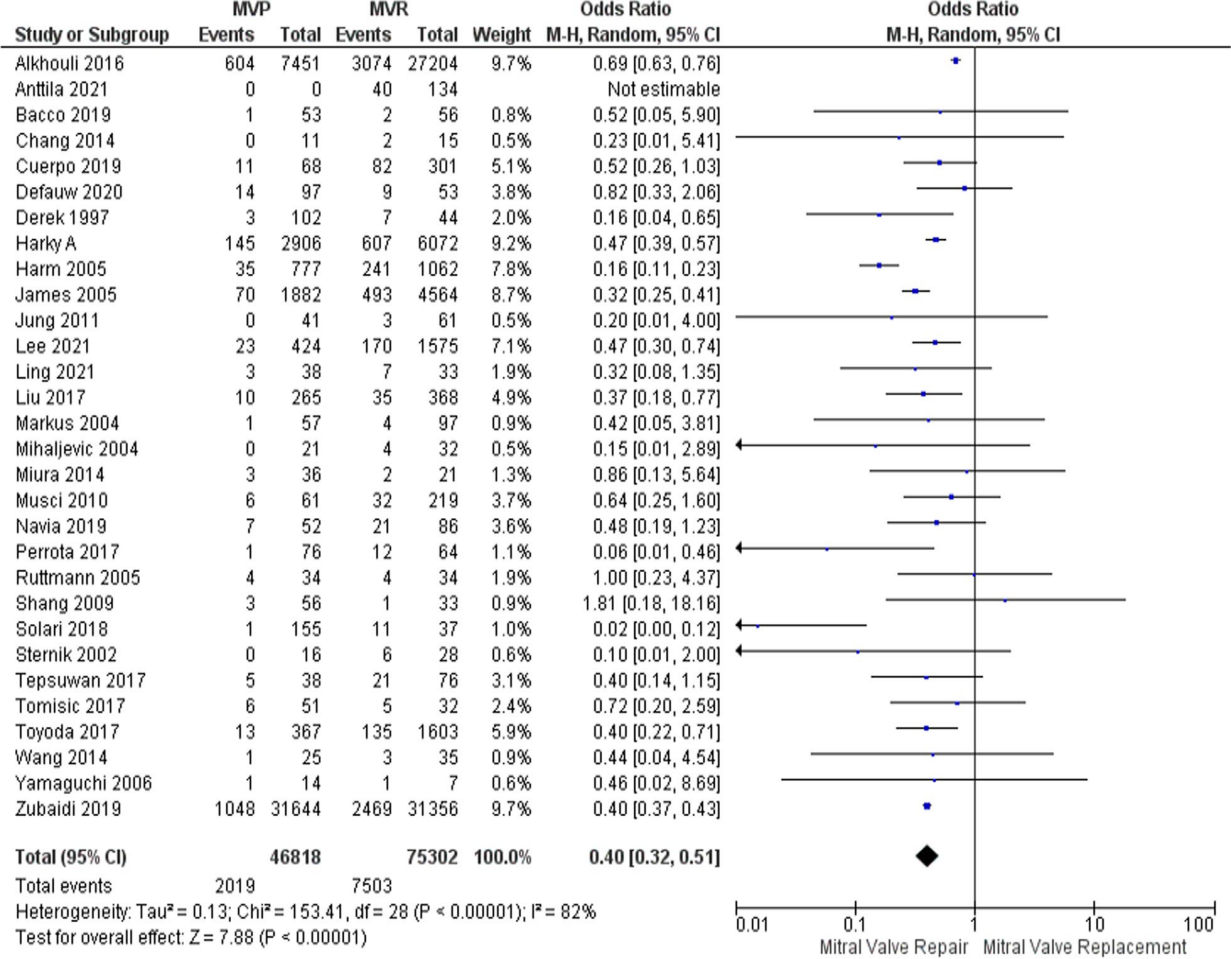
Forest plot for outcome of mortality rates

Out of the 32 studies that were analyzed, the data from 27 of them were available for evaluating survival outcomes. Using a random effects model, the analysis demonstrated that individuals who underwent MVP had a reduced long-term risk of mortality (OR was 0.40, the 95% CI was 0.32–0.51, and *p* = 0.001, with an *I*^2^ of 81%, as shown in Fig. 3). To address the heterogeneity, a sensitivity analysis was performed using the leave-one-out method. Even when influential studies were excluded, the analysis still showed a significant association, with an OR of 0.42, 95% CI: 0.35–0.50, and *p* = 0.00001 (Supplementary Figure S6).

### Recurrent endocarditis

**Fig. 4:**
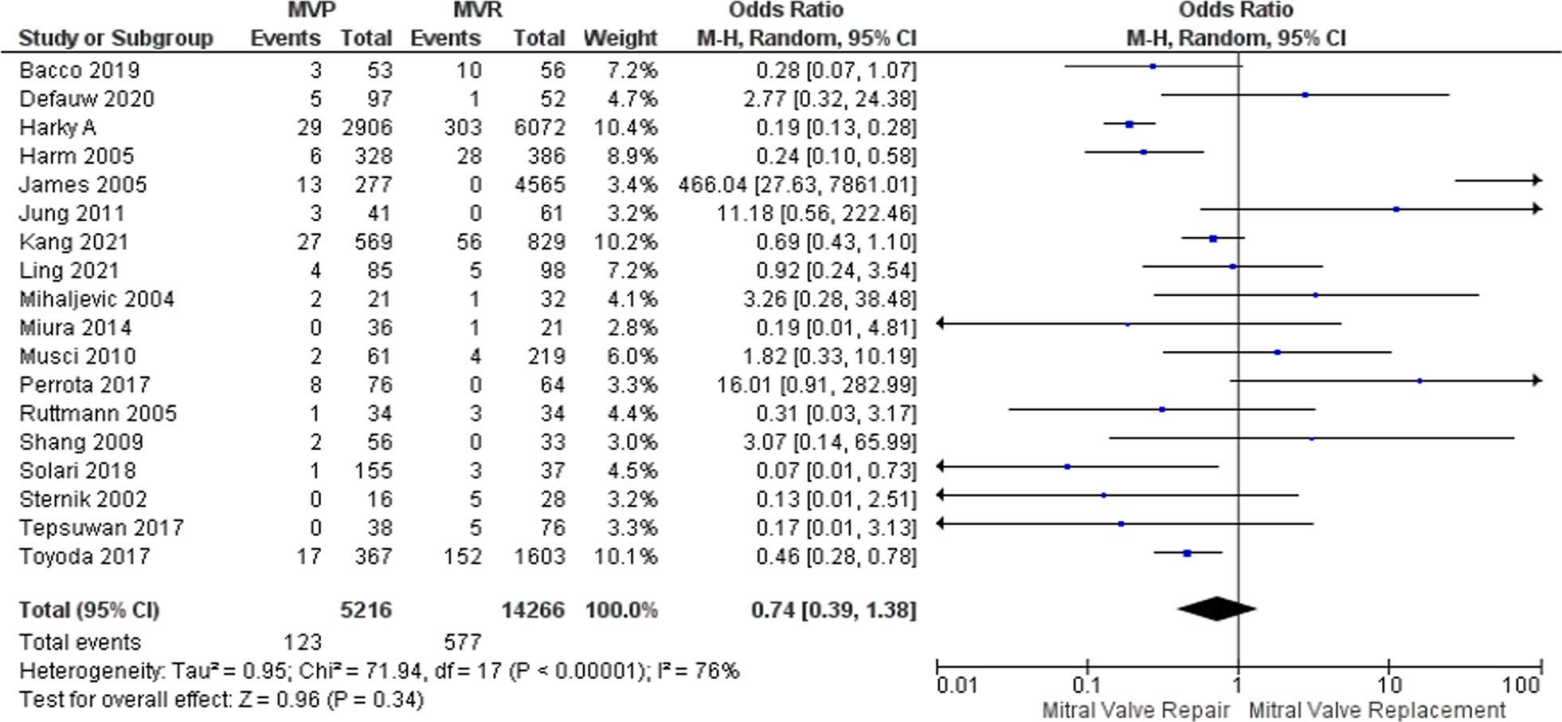
Forest plot for outcome of recurrent endocarditis

Among the 32 studies that were examined, 27 studies involving 5465 patients were utilized to provide data on recurrent endocarditis. Using a random effects model, the analysis indicated that individuals who underwent MVP might have a decreased recurrent endocarditis (OR: 0.75, 95%, CI: 0.39–1.38, *p* = 0.34, *I*^2^ = 76%, as illustrated in Fig. 4). To address the heterogeneity, a sensitivity analysis using the leave-one-out method was performed. Upon excluding influential studies, the analysis yielded an OR of 0.60, with a narrower 95% CI of 0.37–0.97 and reduced heterogeneity (I^2^ = 38%, *p* = 0.04; Supplementary Figure S7).

### Postoperative stroke

**Fig. 5:**
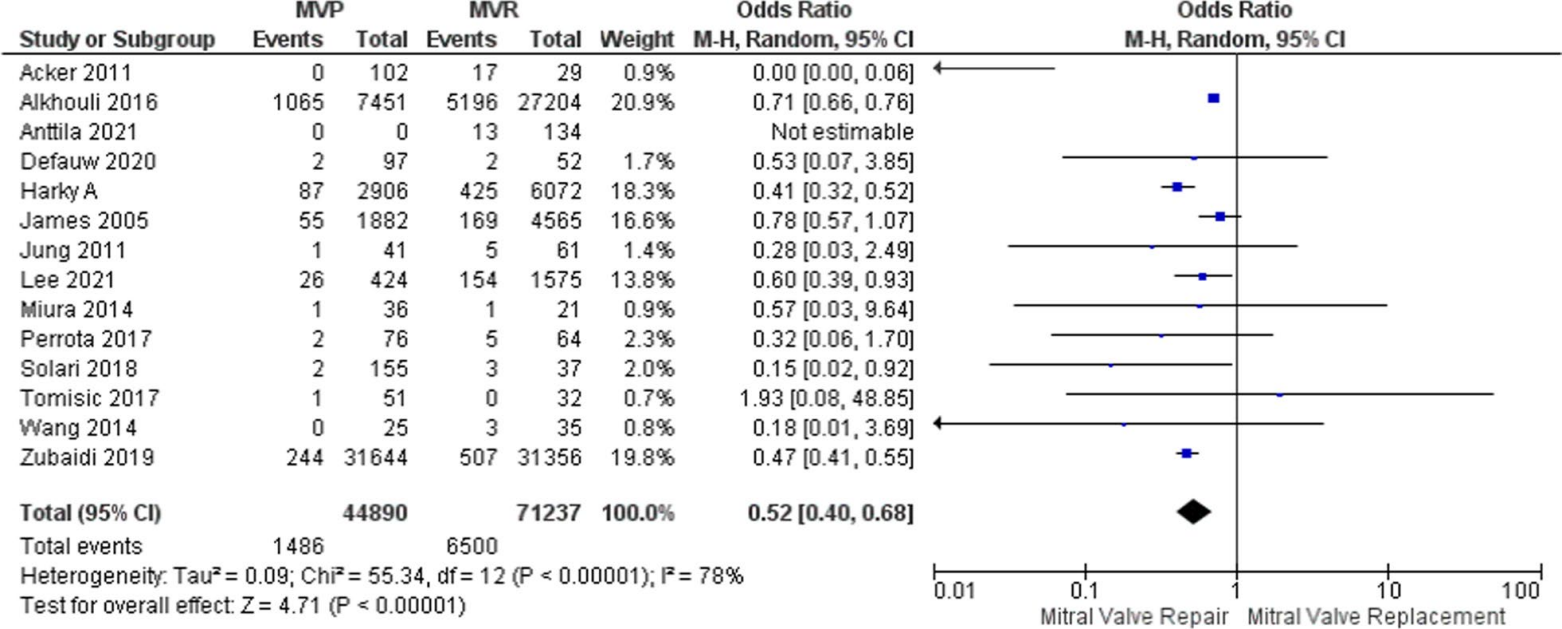
Forest plot for outcome of postoperative stroke

Among the 32 studies included in the analysis, only 10 were used to assess postoperative stroke outcomes. By employing a random effects model, it was discovered that individuals who underwent MVP had a lower likelihood of experiencing postoperative stroke, as demonstrated by an OR of 0.52, 95% CI of 0.40–0.68, and a *p*-value of 0.001, with an *I*^2^ of 78% (Fig. 5). To tackle the issue of heterogeneity, a sensitivity analysis was conducted using the leave-one-out method. Upon excluding influential studies, the analysis revealed an OR of 0.46, a narrower 95% CI of 0.41–0.52, and no observed heterogeneity (*I*^2^ = 0%, and *p* < 0.00001; Supplementary Figure S8).

## Discussion

IE poses a formidable challenge in clinical practice, often requiring surgical intervention to improve patient outcomes. When addressing IE, the choice between MVr or MVP and MVR becomes pivotal. This meta-analysis aims to compare the efficacy of MVP and MVR specifically in the context of treating IE. Understanding the relative effectiveness of these approaches is crucial for clinicians grappling with treatment decisions in the face of increasing IE incidence and the imperative for surgical intervention in severe cases. By synthesizing data from various studies, this analysis endeavors to shed light on outcomes such as postoperative bleeding, mortality rates, recurrent endocarditis, and stroke, thereby elucidating the advantages and drawbacks associated with both MVP and MVR.

A comprehensive meta-analysis examined 32 studies encompassing 82,123 patients to compare the outcomes of MVP and MVR in terms of postoperative bleeding, mortality, recurrent endocarditis, and stroke. The findings revealed that MVP was significantly associated with lower rates of postoperative bleeding compared to MVR. Furthermore, patients who underwent MVP demonstrated a markedly reduced risk of long-term mortality in contrast to those who had MVR. Interestingly, MVR showed lower rates of recurrent endocarditis. Additionally, MVP was linked to a decreased incidence of postoperative stroke. The robustness of these results was confirmed through thorough sensitivity analyses, highlighting the potential advantages of MVP over MVR in reducing postoperative complications and enhancing patient outcomes. A separate meta-analysis indicated that MVP is correlated with considerably lower risks of both early and long-term mortality in comparison with MVR. This study also found that MVP was associated with a reduced risk of recurrent IE. However, no significant disparities were observed between the two procedures regarding the need for re-operations. Despite repair being considered a more complex surgery in IE, similar to the findings in this study, it appears to be more effective than replacing the mitral valve in cases of IE.

The finding that MVP is linked to a lower incidence of postoperative bleeding compared to MVR is a crucial discovery with substantial clinical ramifications. This disparity can be explained by various aspects intrinsic to the surgical methods and the nature of the procedures. MVP, being a more conservative technique that retains the patient's original valve tissue, typically results in less surgical trauma and reduced need for extensive suturing or prosthetic implantation [48, 61, 62]. This preservation of native tissue diminishes the risk of bleeding in comparison with MVR, which involves excising the native valve and substituting it with a prosthetic one. The replacement procedure often necessitates more extensive suturing and manipulation of surrounding tissues, potentially increasing the likelihood of postoperative bleeding [40, 63]. Moreover, the materials employed in MVR, particularly mechanical valves, may require long-term anticoagulation therapy. This anticoagulation elevates the risk of bleeding postoperatively, especially during the early recovery phase. Conversely, MVP frequently does not demand the same level of anticoagulation, particularly when the repair is successful and valve function is adequately restored.

During the early stages of infective endocarditis, patients often encounter significant health issues, and performing reconstructive surgery on inflamed tissue can be extremely difficult. The likelihood of repairing infected mitral valves during acute endocarditis ranges from 30 to 80% [45, 54, 59, 64]. This variability is attributed to differences in demographics and the surgeon's level of expertise in mitral valve surgery, particularly when deciding between repair and replacement [65]. Mitral valve repair is a suitable option when valve damage is limited. A study demonstrated the lasting effectiveness of mitral valve repair, showing a significant freedom from reoperation and reinfection when the remaining valvular tissue could be reconstructed [45]. Previous research has emphasized the crucial role of preserving tissue in achieving successful MV repair, highlighting the importance of prompt intervention in ensuring valve reparability [66, 67]. The patients in the MVP were typically older and required more emergency operations, in addition to receiving more frequent and elevated doses of inotropic support. Moreover, they demonstrated a higher incidence of severe cardiac decompensation [32, 46, 48].

Research indicates that mitral valve replacement is typically considered for patients who are in the most critical condition and for whom mitral valve repair is not feasible [68]. Consequently, it is expected that these patients may experience poorer postoperative outcomes. Moreover, in the previous studies, the decision between mitral valve repair and replacement may have been influenced by surgeon preference or their experience with valve repair techniques [68–70].

A study by Mikus et al. [63] examined the causative organisms of infective endocarditis rather than surgical techniques for its treatment. Their research indicates that MVP is typically favored for degenerative conditions. The investigation revealed that the choice of surgical approach does not substantially impact major postoperative complications, mortality, or medium-to-long-term survival in IE cases. However, a trend toward higher relapse rates with MVP was observed, potentially due to the large number of cases with unidentified microbial causes, resulting in non-specific antimicrobial therapy. Importantly, the study emphasizes that infections caused by Staphylococcus aureus and coagulase-negative staphylococci are linked to elevated risks of early mortality and relapse [63, 71]. Despite these concerns, MVP may still be a suitable option when it ensures complete eradication of infected tissue, particularly in cases involving Streptococcus infections, younger patients, and following a comprehensive course of antibiotic treatment lasting at least 18 days. Therefore, ensuring appropriate antibiotic coverage is crucial regardless of the surgical approach to optimize outcomes.

The observed decline in bleeding rates linked with MVP not only suggests potential benefits in terms of decreased surgical complications but also indicates improved patient outcomes and shorter recovery periods. While both procedures aim to tackle mitral valve disease and enhance patient well-being, the reduced risk of postoperative hemorrhage associated with MVP may sway the decision in its favor for certain individuals. Nonetheless, it is crucial to consider individual patient variables such as the severity of valve pathology, overall health condition, and surgical proficiency when determining the appropriate treatment approach. The fundamental goal of endocarditis surgery is to entirely eliminate infection and reinstate the functional anatomy of the affected valve [39].

The results of this meta-analysis have significant clinical implications. Although both MVR and MVP are viable options for treating mitral valve disease, MVP appears to offer several advantages in terms of postoperative bleeding, long-term mortality, and stroke risk. However, it is crucial to consider individual patient factors, such as the extent of valve damage, comorbidities, and surgical expertise, when making treatment decisions. Only surgeons with extensive experience and adequate skills are capable of repairing severely damaged mitral valves [52]. Furthermore, no guidelines have been established for repairs in infected valves, which are much more complicated. The ratio and underlying patients of MVP varied greatly among different surgical teams and centers [72].

When comparing MVP and MVR in cases of IE, it is crucial to acknowledge the inherent selection bias. The choice between repair and replacement is often influenced by the endocarditis severity and the surgical team's specific expertise. Typically, patients chosen for valve repair have less severe cases, while those undergoing replacement may have more advanced or complex endocarditis. This disparity in patient selection can impact the comparability of outcomes between the two groups. Moreover, surgical teams with extensive experience in MVR may achieve superior results due to their specialized skills, highlighting the significance of surgical training in repair techniques. Although these biases and challenges in external reproducibility must be recognized, they should not discourage surgeons from pursuing training in valve repair. Developing expertise in this area remains essential for improving patient outcomes.

It is crucial to recognize the limitations of this meta-analysis. To begin with, incorporating studies with limited participant numbers may have introduced a higher degree of selection bias, potentially affecting the broader applicability of the results. Furthermore, the range of methods and techniques used for MVP across different studies, often influenced by individual surgical expertise, could result in outcome variability. Moreover, the absence of a direct comparison between MVP and MVR in patients with resolved IE restricts our capacity to conclusively evaluate the comparative efficacy of these interventions in this particular group. The diversity in study methodologies, patient characteristics, and surgical approaches may also impact result interpretation. Additional research, particularly through randomized controlled trials, is necessary to confirm these findings and establish more robust evidence for clinical decision making. Future studies should also conduct separate analyses for mechanical and bioprosthetic valves to gain more comprehensive insights into the specific outcomes associated with each type of valve replacement. The studies have not reported the proportion of intravenous drug users within each patient cohort, which could have influenced both short-term and long-term outcomes regardless of the surgical procedure employed. It is worth noting that mitral valve replacement is typically reserved for the most severely ill patients.

## Conclusion

In conclusion, our meta-analysis indicates that MVP demonstrates clear advantages over MVR in treating IE when repair is feasible. MVP is associated with lower rates of postoperative bleeding, reduced long-term mortality, and decreased risk of postoperative stroke. Additionally, MVP is linked to a lower risk of recurrence compared to MVR. These findings underscore the potential benefits of MVP in improving patient outcomes. However, further research, including randomized controlled trials, is needed to validate these results and refine treatment strategies for IE management. Individualized decision making based on patient characteristics and surgical expertise remains crucial in optimizing outcomes for IE patients.

## Supplementary Information

Supplementary Material 1: Supplementary Figure S5: Postoperative Bleeding Supplementary Figure S6: Mortality Supplementary Figure S7: Recurrent endocarditis Supplementary Figure S8: Postoperative Stroke Supplementary Figure S9: Funnel plots for Postoperative Bleeding Supplementary Figure S10: Funnel plots for Mortality Supplementary Figure S11: Funnel plots for Recurrent Endocarditis Supplementary Figure S12: Funnel plots for post-operative stroke.

Supplementary Material 2: Supplementary Table 1: Newcastle Ottawa Quality Assessment scale.

## Data Availability

Data are available within the article. The authors confirm that the data supporting the findings of this study are available within the article.

## Abbreviations

IE: Infective endocarditis
MVP: Mitral valve repair or plasty
MVR: Mitral valve replacement
ICU: Intensive care unit
PRISMA: Preferred Reporting Items for Systematic Review and Meta-Analysis
OR: Odds ratio
CI: Confidence interval
