# Supplementary material for: Mitral valve repair and replacement in infectious endocarditis: a systematic review and meta-analysis of clinical outcome
Source: Egypt Heart J. 2024 Oct 4;76:134. doi: 10.1186/s43044-024-00564-5 (PMC11452577; doi:10.1186/s43044-024-00564-5)
Supplement: Supplementary file 1 — Supplementary Material 1: Supplementary Figure S5: Postoperative Bleeding Supplementary Figure S6: Mortality Supplementary Figure S7: Recurrent endocarditis Supplementary Figure S8: Postoperative Stroke Supplementary Figure S9: Funnel plots for Postoperative Bleeding Supplementary Figure S10: Funnel plots for Mortality Supplementary Figure S11: Funnel plots for Recurrent Endocarditis Supplementary Figure S12: Funnel plots for post-operative stroke. [file 43044_2024_564_MOESM1_ESM.docx]

| **First author** | **Newcastle-Ottawa Quality Assessment scale** | | | | | | | | | | **Total score** | **Quality** |
| --- | --- | --- | --- | --- | --- | --- | --- | --- | --- | --- | --- | --- |
|  | **Selection** | | | | | **Comparibility** | | **Outcome** | | |  |  |
| Bacco 2019 | * | * | * | * | / | | / | * | * | * | 7 | Good |
| Ferringa HH 2005 | * | * | * |  | * | | * | / | * | * | 7 | Good |
| Alkhouli 2019 | / | / | * | * | * | | * |  | * | * | 6 | Fair |
| Lee 2021 | / | / | * | * | * | | / | * | * | * | 6 | Fair |
| Tepsuwan 2017 | * | * | * | * | * | | * | / | * | * | 8 | Good |
| Malvindi 2021 | * | * | * | * | * | | * | * | * | * | 9 | Good |
| Harky 2017 | * | * | * | * | * | | / | * | * | * | 8 | Good |
| Ling C.N.Y 2021 | * | * | * | / | * | | * | / | * | / | 6 | Good |
| Zubaidi 2019 | * | * | * | * | * | | * | * | * | * | 9 | Good |
| Kang 2021 | * | * | * | * | * | | / | * | / | * | 7 | Good |
| Liu J.Z 2018 | * | * | * | * | * | | * | * | * | * | 9 | Good |
| Anttila 2021 | * | * | * | * | * | | / | * | * | * | 8 | Good |
| Chang 2014 | * | * | * | * | * | | / | * | * | * | 8 | Good |
| Solari 2018 | * | * | * | * | * | | / | * | * | * | 8 | Good |
| Perotta 2017 | * | * | * | * | * | | / | * | * | * | 8 | Good |
| Cuerpo 2019 | * | * | * | * | * | | / | * | * | * | 8 | Good |
| Jung 2011 | * | * | * | * | / | | / | * | * | * | 7 | Good |
| Miura 2014 | * | * | * | * | * | | / | * | * | * | 8 | Good |
| Musci 2010 | * | * | * | * | * | | * | * | * | * | 9 | Good |
| Mihaljevic 2004 | * | * | * | * | * | | / | * | * | * | 8 | Good |
| Sternik 2002 | * | * | * | * | * | | / | * | * | * | 8 | Good |
| Shang 2009 | * | * | * | * | * | | * | * | * | * | 9 | Good |
| Tomisic 2017 | * | * | * | / | * | | * | * | * | * | 8 | Good |
| Defauw 2020 | * | * | * | / | * | | * | * | * | * | 8 | Good |
| Navia 2019 | * | * | * | * | * | | / | * | * | / | 7 | Good |
| Wang 2014 | * | * | * | / | * | | * | * | * | * | 8 | Good |
| Yamaguchi 2006 | * | / | * | / | * | | / | * | * | * | 6 | Fair |
| Toyoda 2017 | * | * | * | * | * | | * | / | * | * | 8 | Good |
| Ruttman 2005 | * | * | * | / | * | | / | * | * | * | 7 | Good |
| Wilhelm 2004 | * | * | * | * | * | | * | * | * | / | 8 | Good |
| Derek D.D 1997 | * | * | * | / | * | | / | * | * | * | 7 | Good |
| James 2005 | * | * | * | * | * | | * | * | * | * | 9 | Good |
