# Supplementary material for: Mitral valve repair and replacement in infectious endocarditis: a systematic review and meta-analysis of clinical outcome
Source: Egypt Heart J. 2024 Oct 4;76:134. doi: 10.1186/s43044-024-00564-5 (PMC11452577; doi:10.1186/s43044-024-00564-5)

**Supplementary Figure S5: Postoperative Bleeding**


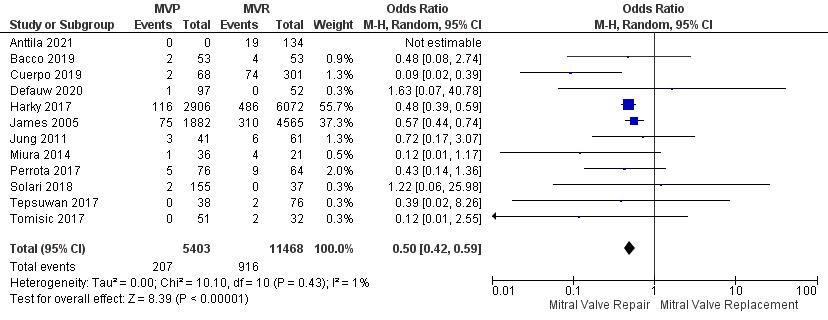


**Supplementary Figure S6: Mortality**


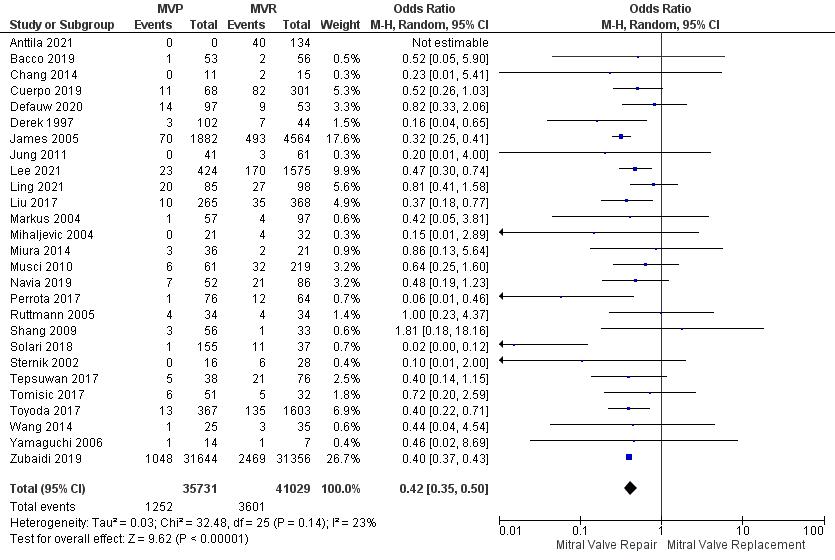


**Supplementary Figure S7: Recurrent endocarditis**

**
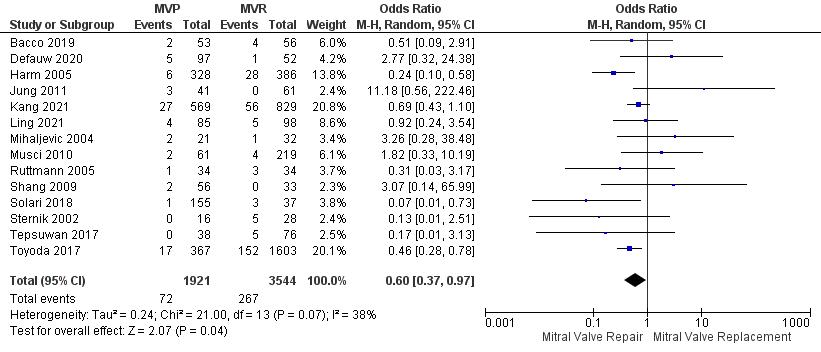
**

**Supplementary Figure S8: Postoperative Stroke**

**
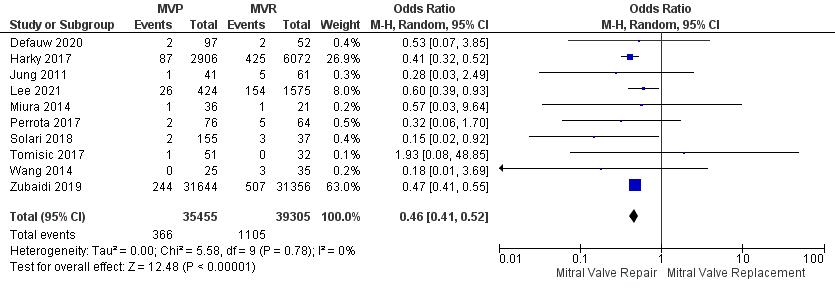
**

**Supplementary Figure S9: Funnel plots for Postoperative Bleeding**


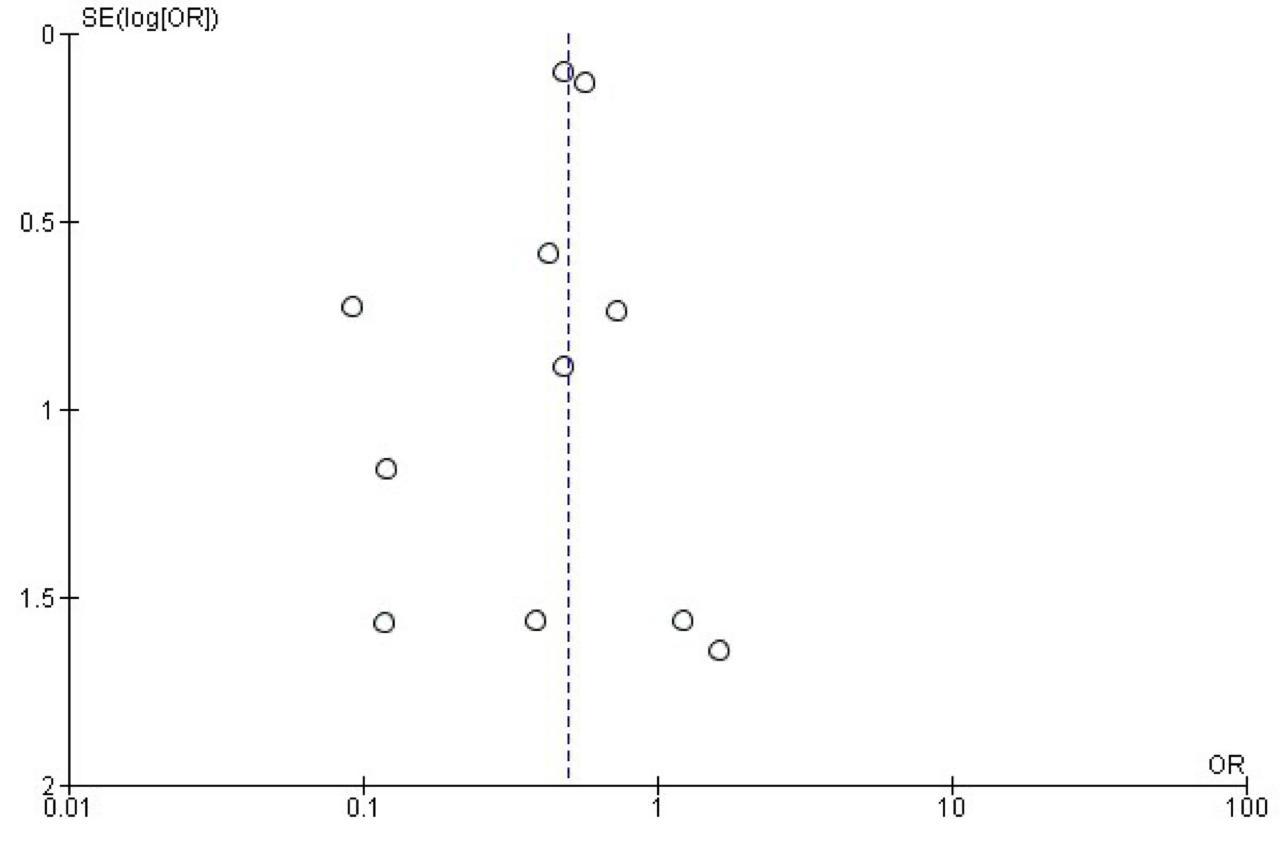


**Supplementary Figure S10: Funnel plots for Mortality**


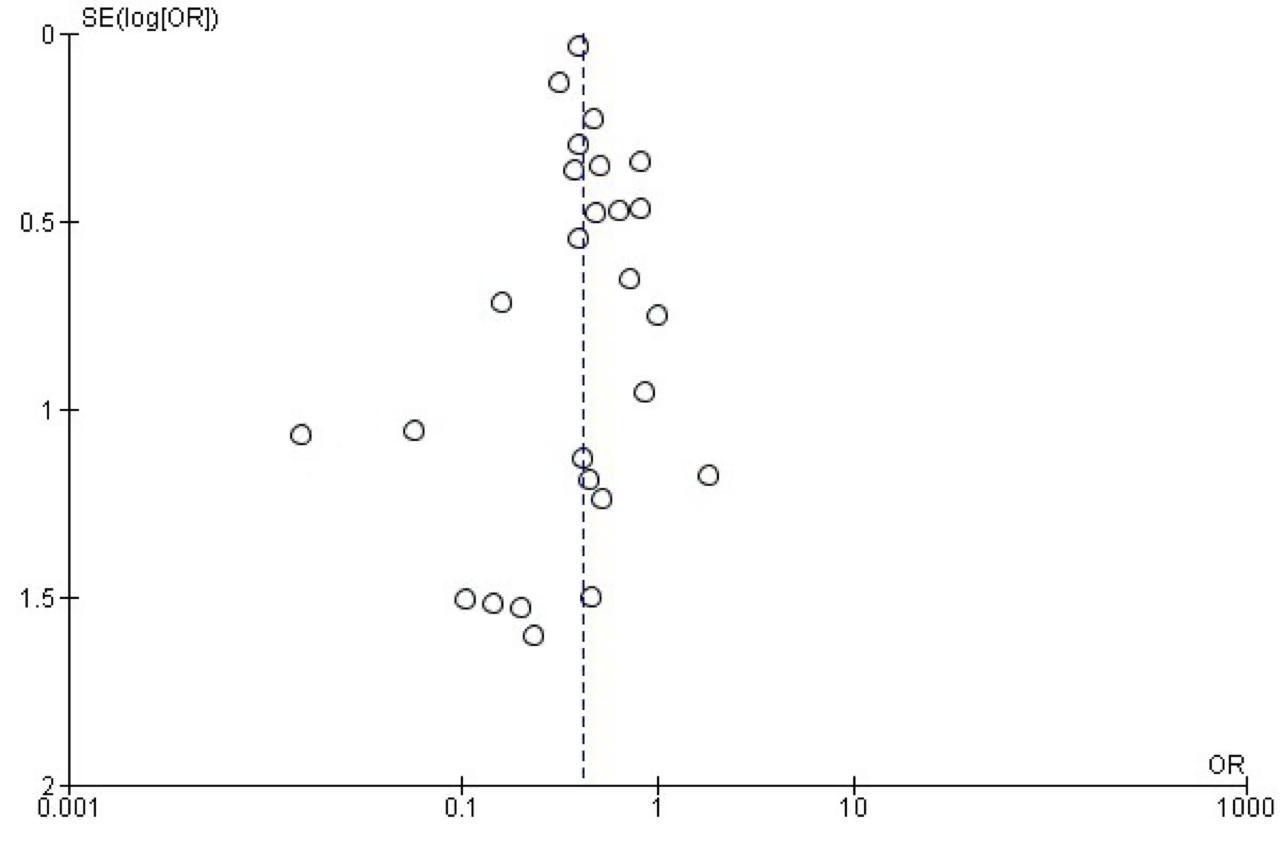


**Supplementary Figure S11: Funnel plots for Recurrent Endocarditis**


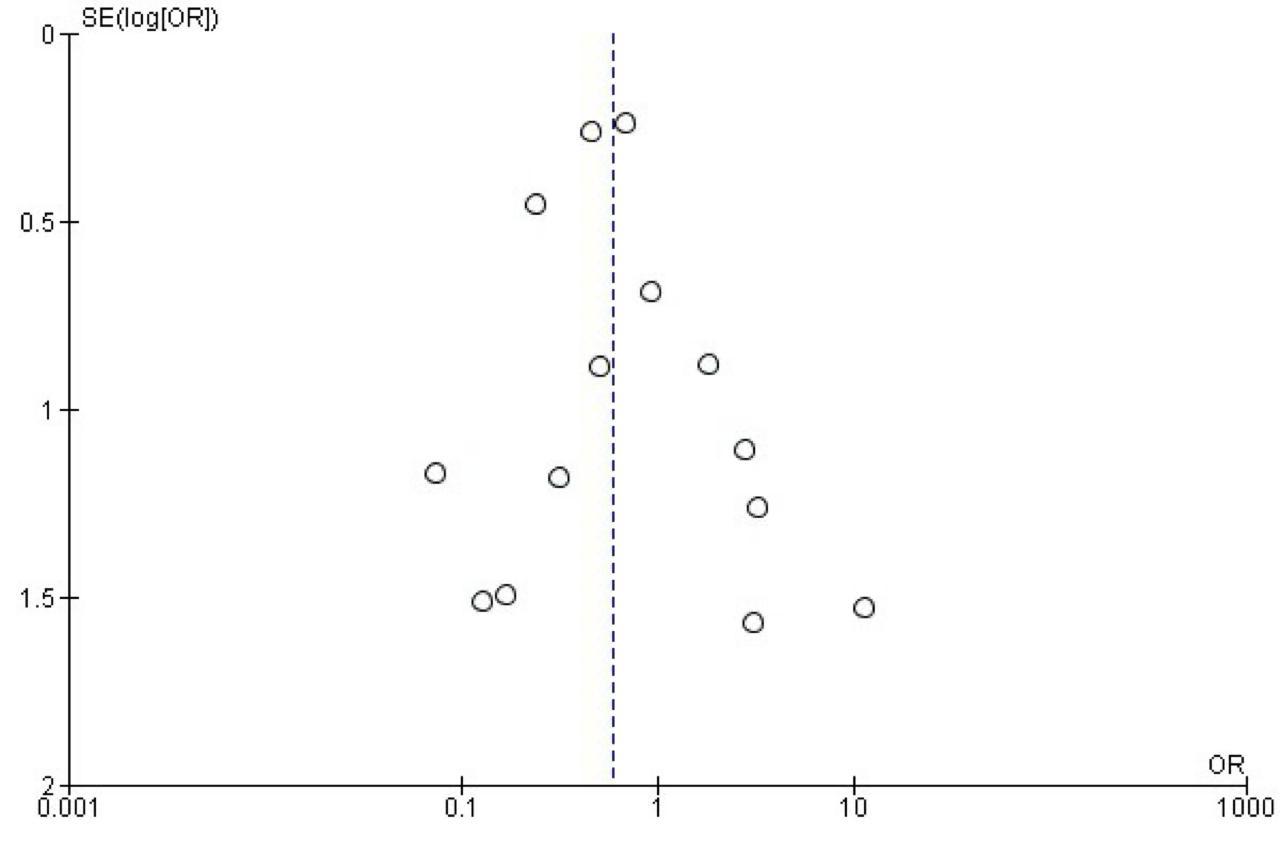


**Supplementary Figure S12: Funnel plots for Post-operative stroke**


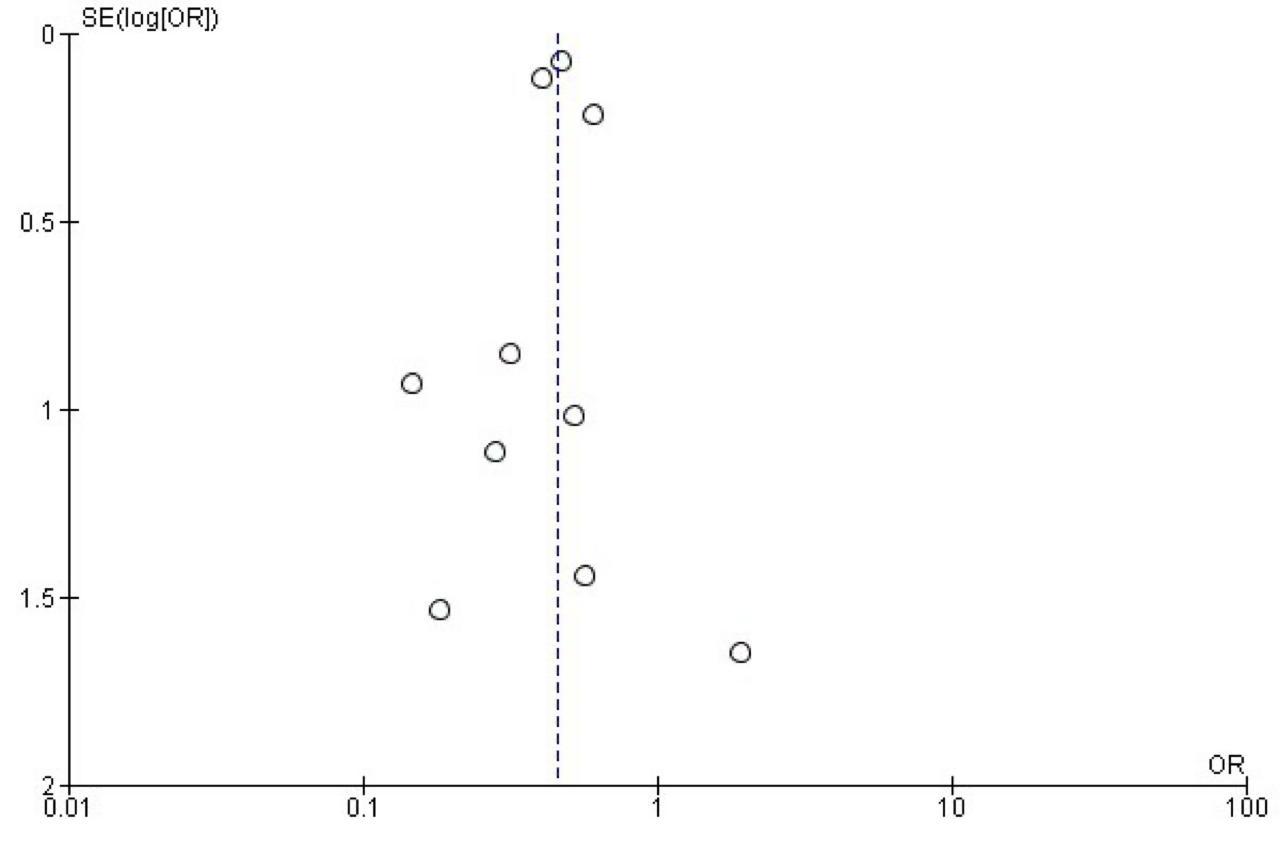

Supplement: Supplementary file 2 — Supplementary Material 2: Supplementary Table 1: Newcastle Ottawa Quality Assessment scale. [file 43044_2024_564_MOESM2_ESM.docx]
